# Late-onset chylothorax during chemotherapy after lobectomy for lung cancer: A case report and review of the literature

**DOI:** 10.1097/MD.0000000000015909

**Published:** 2019-05-31

**Authors:** Chu Zhang, Rui-Mei Zhang, Yong Pan, Wen-Bin Wu, Miao Zhang

**Affiliations:** a Department of Thoracic Surgery, Shaoxing People's Hospital (Shaoxing Hospital of Zhejiang University), Shaoxing; b Department of General Surgery, Xuzhou Infectious Disease Hospital; c Department of Thoracic Surgery, Xuzhou Central Hospital Affiliated to Southeast University, Xuzhou, China.

**Keywords:** chylothorax, iatrogenic, late-onset, single-direction, spontaneous, uniportal

## Abstract

**Rationale:**

Chylothorax is usually diagnosed within a few days after lobectomy. Late-onset chylothorax following trauma or thoracic surgery is rare but potentially lethal, lacking reliable preventive methods.

**Patient concerns:**

A 54-year-old male patient complained of dyspnea during adjuvant chemotherapy on the 35th postoperative day after right middle lobectomy and systemic lymph node dissection (SLND) for lung cancer. His computed tomography indicated massive pleural effusion filling in the right chest cavity.

**Diagnoses:**

The patient was primarily diagnosed as late-onset chylothorax, without definite evidence to exclude spontaneous chylous leakage.

**Interventions:**

Uniportal video-assisted thoracoscopic thoracic duct ligation (TDL) was performed for him, as conservative treatment using octreotide with fat-free diet turned out to be ineffective.

**Outcomes:**

His pleural effusion was gradually diminished after reoperation, and the patient was discharged 9 days after TDL.

**Lessons:**

Postoperative late-onset or spontaneous chylothorax should be kept in mind after pulmonary resection and SLND, and the exclusion of chylous leakage could be considered as a precondition of chest tube removal.

## 1 Introduction

Chylothorax following lobectomy and systemic lymph node dissection (SLND) is a rare but potentially lethal complication, which is ascribed to injury of thoracic duct or lymphatic tributaries.^[1]^ Chylothorax is potentially lethal if not timely diagnosed and effectively treated. The etiology of chylothorax can be simply classified into spontaneous and traumatic (injury or surgery). A spontaneous chylothorax may be congenital, infectious, neoplastic, or due to unknown conditions that can cause obstruction of the thoracic duct or aberrant lymphatic flow.^[2]^ The most common cause of traumatic chylothorax is thoracic surgery after neoadjuvant chemoradiotherapy.^[3]^

Chylous leakage leads to malnutrition, immunosuppression, and respiratory distress through the loss of chylous fluid,^[4]^ which could be initially treated with fat-free or low-fat diet and administration of somatostatin. Failure after conservative treatment necessitates surgical intervention such as thoracic duct ligation (TDL) or resection. However, surgical management is not always effective.^[4]^

Most instances of postoperative chylothorax are diagnosed within 3 days following anatomic pulmonary resection. To the best of our knowledge, delayed chylous leakage following thoracic surgery is uncommon. Herein, a late-onset chylothorax in a patient nearly 1 month after lobectomy and SLND was presented, followed by a brief review of related literatures.

## 2 Case presentation

**Figure 1:**
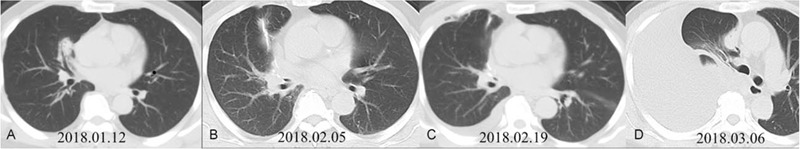
(A) CT scan on his first admission showed pulmonary nodule located in right middle lobe. (B) CT on 12th POD after chest tube removal indicated re-expansion of right upper and lower lobes, without significant PE. (C) CT on 26th POD showed mild-to-moderate right-sided PE. (D) CT on 40th POD showed that his right chest cavity was filled with massive PE. CT = computed tomography, POD = postoperative day, PE = pleural effusion.

This report was approved by Institutional Review Board of Xuzhou Central Hospital, and written informed consent was obtained from the patient. The clinical data were treated anonymously. A 54-year-old male patient was presented with persistent cough and sputum for 2 months on January 24, 2018, without hemoptysis, hoarseness, chest distress, thorcalgia, dorsalgia, or significant weight loss during this period. He denied previous disease history such as tuberculosis, bronchiectasis, rheumatism, or lymphoma, except a smoking history of 2.5 pack-years. Physical examination, laboratory tests including carcinoembryonic antigen, neuron-specific enolase, squamous cell carcinoma antigen, and cytokeratin-19 fragment showed nothing abnormal. Empirical antibiotics had not been used because repeated culture of his sputum was negative. Further radiological examinations were performed step by step for a clinical diagnosis. A chest computed tomography (CT) was carried out, and a pulmonary nodule measuring 1.5 cm × 2.0 cm was indicated in his right middle lobe (Fig. 1A), in suspicious of malignancy. However, a biopsy under bronchoscopy failed to deliver a definite diagnosis. Distal metastasis or other disease was excluded by further abdomen CT, cranial magnetic resonance, and whole-body bone emission CT. Then it was staged as cT1bN0M0 (IA2) according to the 8th edition of TNM staging system for lung cancer. After a multidisciplinary evaluation, minimally invasive thoracic surgery was decided for this patient.

Fast-track protocol in thoracic surgery was utilized. Oral carbohydrate-containing clear fluid was administered 6 hours (400 mL) and 2 hours (200 mL) before the operation, respectively. His right-sided pulmonary arterial tree was reconstructed using three-dimensional computed tomography (3D-CT) by software OsiriX on Macintosh platform (Apple, Cupertino, CA). Preoperative simulation of anatomical pulmonary resection was performed using the 3D model, with the aim to diminish accidental injury of abnormal vessels. Thereafter, right middle lobectomy was carried out by uniportal video-assisted thoracoscopic surgery (VATS), in a single direction from the front to the back of the patient. It followed the sequence of right middle pulmonary vein, oblique fissure, lateral segmental artery, lobar bronchus, medial segmental artery, and the horizontal fissure. SLND was finally manipulated as frozen section of the tumor showed a diagnosis of lung cancer. Prophylactic ligation of the thoracic duct, mechanical or chemical pleurodesis was not performed initially. Patient-controlled analgesia and ultrasound-guided serratus anterior plane block were applied to improve pain relief and to promote mobilization out of bed after surgery.^[5]^ One 26 French chest tube was inserted for chest drainage. Nausea or vomiting was controlled efficiently. His postoperative pathological diagnosis was pulmonary adenocarcinoma with visceral pleura invasion (pT2aN0M0, IB). His chest tube was removed on the 3rd postoperative day (POD), without a chyle test of the chest drainage. Then he was discharged uneventfully. On the 12th POD, his chest CT showed satisfactory re-expansion of right upper and lower lobes without significant pleural effusion (Fig. 1B).

Nearly 1 month later, he was readmitted for adjuvant chemotherapy, without palpitation, cough, dyspnea, or fever, except mild tightness of his right chest. However, the physical examination indicated generally normal respiratory sound. Another CT indicated mild-to-moderate volume of pleural effusion in his right chest cavity (Fig. 1C). Nevertheless, a chest tube was not re-inserted timely, because it was empirically misdiagnosed as hypoproteinemia rather than haemothorax or chylothorax by the clinician. As his laboratory test showed normal blood cell counts, adjuvant pemetrexed (500 mg/m^2^ of body surface area) and cisplatin (75 mg/m^2^ of body surface area) was conducted. On the 2nd day of chemotherapy, he complained of nausea and gradually worsened respiratory distress, without vomiting. But he was also misdiagnosed as pulmonary edema associated with inadequate hydration during the administration of cisplatin. Nevertheless, his suffering was not alleviated after diuretic therapy using 40 mg of furosemide. On the next day, he complained of chest tightness, dysphagia, and intolerable dyspnea.

**Figure 2:**
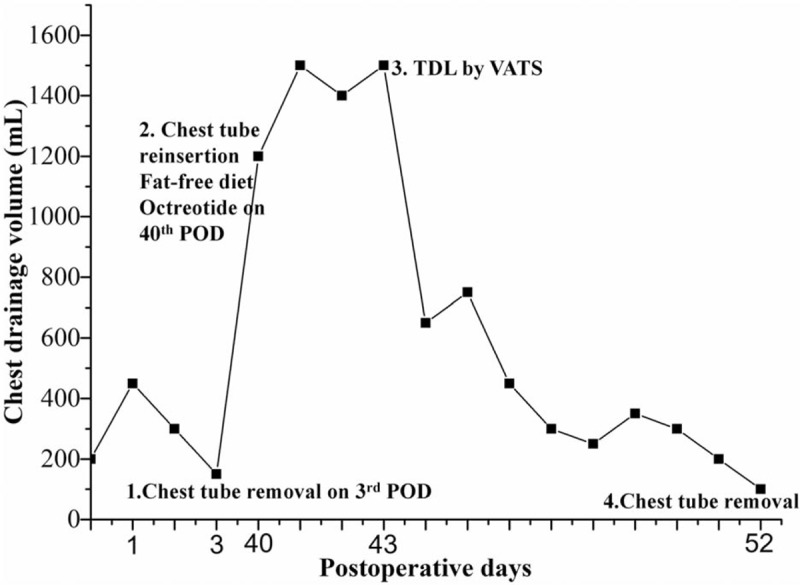
Chylous drainage trend and treatment of the patient. TDL = thoracic duct ligation.

Physical examination showed that his right-sided breath sound was absent, and his chest CT indicated massive pleural effusion filling in his right chest cavity, which was suspicious of late-onset severe chylothorax (Fig. 1D). Then a 28-French chest tube was inserted, along with octreotide (0.1 mg every 8 hours) and fat-free diet. The fluid appeared milky, and chylothorax was confirmed by positive staining of chyle. Because his daily chest drainage was >1 L for another 3 days, a uniportal VATS examination under general anesthesia was chosen. Multiple band ligations of the thoracic duct were performed, and his chest drainage was decreased during the next 9 days. The chylous drainage trend was illustrated as Fig. 2. When the chest radiography revealed fully expanded pulmonary lobes, he was discharged after catheter removal. During the follow up of 3 months, his chest radiography showed nothing abnormal and the patient demonstrated satisfactory quality of life.

## 3 Discussion

Iatrogenic chylothorax ranges from 1.4% to 2.3% after lung resection and SLND, with higher incidence in N2 disease and those who underwent robotic resection.^[6,7]^ Another study indicates that the overall incidence of chylothorax following lobectomy and pneumonectomy is 1.04%, moreover, the operation site, resection type, technique of SLND, histology, and staging of the lung cancer do not influence the occurrence of postoperative chylothorax.^[8]^ Additional factors associated with a relatively high rate of chylothorax include subclavian vein catheterization, mediastinal tumor resection, thoracic aneurysm repair, and sympathectomy.^[3]^ Patients typically present with dyspnoea, chest pain, cough, and fatigue. Although the chylous effusion is mainly milky, it can also appear serous, sanguineous, or bloody. Therefore, absence of a milky pleural effusion does not exclude the diagnosis of chylous leakage.^[3]^

A timely diagnosis and management of chylothorax is essential. This presented case was considered as late-onset or delayed surgery-related chyle fistula, which was initially misdiagnosed empirically when the pleural effusion was not massive. Previously reported cases of late-onset chylothorax were listed in Table 1. Generally, it could occur in any age (range from 9-year to 79-year old). Besides, the interval between thoracic surgery or trauma and the onset of chylothorax could ranges from less than a week to 20 years. In addition, delayed chylothorax could also result from thoracic irradiation and/or systemic chemotherapy.^[26–28]^ As for the presented case, the severe chyle leakage was noticed during the adjuvant chemotherapy following lobectomy. However, the definite role of chemotherapy on chylothorax is unknown.

**Table 1:**
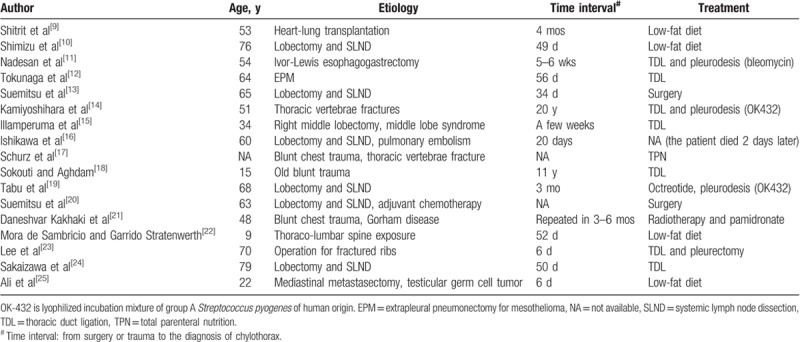
Previously reported cases of late-onset chylothorax after surgery or trauma.

On the other hand, although this presented case didn’t suffer from severe vomiting before the onset of dyspnea, spontaneous chylothorax could not be excluded definitely. Chylothorax can occur in cases of congenital lymphatic malformation such as lymphangiectasis, Noonan syndrome, lymphangioleiomyomatosis (LAM),^[29]^ additionally, it could also be idiopathic.^[30–32]^ Specifically, LAM is a rare disease that affects women in fertile age, mostly involving lung and mediastinic lymph nodes.^[33]^ The pulmonary disease is characterized by dyspnea, pleural effusion, hemoptysis, and spontaneous pneumothorax, while chylothorax appears in up to 30% of cases.^[33]^

The indication of surgical intervention for chylothorax remains uncertain. Patients with chyle flow outputs of >1 L per day should be treated timely and effectively. Besides, if a low-volume chylothorax begins to deteriorate, reoperation could also be indicated. Lymphangiography and lymphoscintigraphy are useful to localize the thoracic duct leakage. If conservative management fails, TDL via VATS is a reasonable choice.^[3,34]^

It is noteworthy that, unplanned readmission for late-onset chylothorax might be underreported. Based on the available data, chylothorax following SLND might be mainly a technical issue, lacking reliable preventive methods. First, there is no consensus on the effect of prophylactic TDL. A review and meta-analysis of English and non-English articles using OVID and MEDLINE (1980–2014) shows that prophylactic TDL could reduce the incidence of postoperative chylothorax following esophagectomy for cancer.^[35]^ However, another meta-analysis indicates that, prophylactic TDL is not useful in reducing the incidence of post-esophagectomy chylous leakage.^[36]^ Second, lobectomy using fast-tracking program could deliver reduced postoperative stay and rapid discharge,^[37]^ nevertheless, another review shows that length of stay after lobectomy is negatively associated with unplanned readmission of the patients.^[38]^ The incidence of chylothorax might be correlated with uniportal VATS procedure for SLND and quick removal of chest tube. Therefore, the addition of proper follow up for unplanned readmission to fast-track protocol might be necessary. In addition, it is reported that mediastinal micro-vessels clipping during SLND may reduce postoperative pleural drainage.^[39]^ Better evidence is warranted.

In summary, a late-onset or spontaneous chylothorax following lobectomy and SLND should be kept in mind when the patient complained of dyspnea, and an exclusion of chyle from chest drainage could be considered as a precondition of chest tube removal. Further studies regarding the prevention of chylous leakage during SLND is warmly welcomed.

## Author contributions

**Conceptualization:** Miao Zhang.

**Data curation:** Chu Zhang, Rui-Mei Zhang, Miao Zhang.

**Formal analysis:** Chu Zhang, Rui-Mei Zhang.

**Funding acquisition:** Rui-Mei Zhang, Yong Pan, Miao Zhang.

**Methodology:** Rui-Mei Zhang, Miao Zhang.

**Resources:** Wen-Bin Wu.

**Validation:** Chu Zhang.

**Writing – original draft:** Chu Zhang, Rui-Mei Zhang, Yong Pan, Wen-Bin Wu, Miao Zhang.

**Writing – review & editing:** Chu Zhang, Yong Pan, Wen-Bin Wu, Miao Zhang.
